# Evaluation of the Efficiency of Biological Treatment in Activated Sludge from a WWTP at Laboratory Scale for the Elimination of Biomicroplastics and Related Products

**DOI:** 10.3390/molecules31111878

**Published:** 2026-05-29

**Authors:** David Alcaide-Benavides, Eloy Torres-Arévalo, Marinella Farré, Marta Llorca

**Affiliations:** 1 Institute of Environmental Assessment and Water Research, C/Jordi Girona, 18-26, 08034 Barcelona, Spain; dabqsh@cid.csic.es (D.A.-B.);; 2 Doctoral Program in Analytical Chemistry and Environmental Science, Department of Chemical Engineering and Analytical Chemistry, University of Barcelona, 08028 Barcelona, Spain

**Keywords:** biomicroplastics, biodegradation in WWTP, Py-GC-HRMS, LC-HRMS, plastic additives, suspect screening

## Abstract

Nowadays, bioplastics are increasingly being used as an alternative to single-use fossil-based plastics. However, a major challenge associated with bioplastics is the need for higher amounts of plastic additives to achieve material properties comparable to those of conventional plastics, which raises concerns regarding their potential ecological impact. In this study, we evaluated the capacity of mixed liquor sludge from a wastewater treatment plant (WWTP) to eliminate bioplastics and their associated plastic additives compared to fossil-based materials. To this end, we exposed three items under controlled laboratory conditions: pure polylactic acid (PLA) pellets, a PLA garbage bag and a conventional fossil-based polyethylene (PE) bag. The study of plastic degradation was carried out by pyrolysis coupled with gas chromatography high-resolution mass spectrometry (Pyr-GC-HRMS). The results show a higher degree of degradation of biobased bags (96.8 ± 4.0%) and PLA pellets (91.3 ± 9.0%), whereas fossil-based bags of PE exhibited negligible degradation (18.3 ± 25.8%). Furthermore, leaching compounds generated during the treatment process were monitored using a suspect screening strategy by means of liquid chromatography coupled with high-resolution mass spectrometry (LC-HRMS). The main results showed that the concentration of several tentatively identified compounds increased after treatment because of the leaching process or because they were degradation products of other previously leached additives. The evaluation of the associated toxicity of these compounds using predicted no-effect concentrations (PNECs) disclosed that these compounds may pose a risk to organisms in receiving waters.

## 1. Introduction

Global plastics production has grown exponentially, rising from 1.7 million tons (Mt) in 1950 [1] to 400.3 Mt in 2022 [2]. This growth is driven by the versatility of plastics, their widespread applications, cost-effectiveness, and lower energy demand during production compared with alternative materials such as glass or metals [3]. However, plastics pose significant environmental challenges due to improper disposal, low degradability, and long-term persistence in the environment, either as plastic materials or as fragmented forms, including micro- and nanoplastics (MNPLs). Primary microplastics (MPLs) (e.g., fibers, tire dust, particles used in cleaners) are directly released into the environment at small sizes, typically below 5 µm. Secondary MPLs, in contrast, are generated through the fragmentation and erosion of larger plastic items once they enter the environment, particularly in aquatic systems, ending in coastal waters. Because of the high resistance to degradation and biodegradation of plastics, these materials constitute 83% of the total marine litter [4]. Their role as vectors for co-occurring contaminants represents an additional concern. Due to the non-polar nature of plastics, these materials can adsorb organic contaminants from aquatic systems and transport them to organisms [5]. This phenomenon is particularly pronounced in the case of nanoplastics (NPLs) because of their higher active surface area and the increased surface irregularities resulting from weathering processes, which enhance sorption capacity [6]. On the other hand, plastics contain a wide range of plastic additives that impart properties such as color, resistance and flexibility among others. These additives are not chemically bonded to the polymeric chains and can, therefore, be readily leached because of weathering processes affecting plastic residues and transferred to marine organisms. Given that smaller particles exhibit higher active surface-area-to-volume ratios, the extent of additive leaching and accumulation is inversely proportional to particle size [6].

In recent years, bioplastics have emerged as a potential environmentally friendly alternative to conventional plastics. Bioplastics are a broad class of materials that may be bio-based, biodegradable, or both, depending on their composition and production process. They offer the potential to reduce dependence on fossil fuels, lower carbon emissions, and mitigate environmental burdens associated with the end-of-life disposal [7,8]. Despite these advantages, bioplastics currently represent only 0.5% of the approximately 400 Mt of annual plastic production. Nevertheless, their production capacity is expected to increase substantially, from 1.81 Mt in 2022 to ca. 7.43 Mt by 2028 [9]. A key challenge associated with bioplastics is the requirement for higher quantities of plastic additives to achieve material properties comparable to those of fossil-based plastics [10,11]. As a result, a greater number of additives may leach into the environment, raising concerns regarding their potential ecological impact [12]. Some bioplastics, such as PLA, can undergo microbial degradation under specific conditions [13]. To be classified as fully biodegradable, at least 90% degradation must be achieved within six months [14]. Moreover, bioplastics production processes can contribute to other potential impacts including eutrophication, human health risks, and ecotoxicity. These effects are associated with the expansion of agricultural land required for biomass feedstocks cultivation, the use of fertilizers and pesticides, fermentation and chemical processing steps, and land-use changes. In addition, increased bioplastic production may compete with food agriculture, raising concerns regarding food availability.

In recent years, the removal and degradation of environmental plastics have been investigated. The biodegradation of certain types of plastics can occur through different pathways, including aerobic and anaerobic processes, enzymatic degradation and photo-biodegradation [15,16]. It is estimated that approximately 90% of MNPLs entering WWTPs are removed because they are retained in the waste-activated sludge [17]. However, further investigation is needed because they may undergo biodegradation rather than simple retention in sludge, potentially generating transformation products that warrant further evaluation. Moreover, current data on MNPLs are based on particle counting and identification of the polymers by spectroscopic methods such as Fourier Transform Infrared Spectroscopy (FTIR). However, the use of other analytical methodologies to quantify NPLs is necessary. In this case, size-exclusion liquid chromatography and high-resolution mass spectrometry (LC(SEC)-HRMS) allow unequivocal identification and quantification of polymers without particle-size limitations [18,19].

In this study, the central objective was to assess the biodegradation of PLA bioplastics, including plastic additives, by a mixed liquor from activated sludge compared to the biodegradation behavior of PE plastic. On one hand, a suspect screening approach by LC-HRMS was applied for the evaluation of plastic additives that may have been leached during biological treatment, while a new method based on pyrolysis-gas chromatography coupled with high-resolution mass spectrometry was developed and used for the detection and quantification of bionanoplastics (bioNPLs).

## 2. Results

### 2.1. Biodegradation Rates of Bio-Based and Fossil-Based Plastics

In this study, we focused on comparing the biodegradation by conventional activated sludge (CAS) of bio-based and fossil-based plastic materials, selecting PLA and PE trash bags as representatives. Additionally, we compared the biodegradation of PLA in two forms: PLA powder and a powdered PLA plastic bag as an example of a highly used commercial product. The results of the biodegradation are shown in Table 1.

### 2.2. Tentative Identification of Plastic Additives Leached During Biodegradation Experiments

To assess plastic additives leached from the plastic during the degradation experiments, plastic, procedural blanks and experimental controls (biotic control without spike) were carried out in parallel. Then, the analysis was performed by LC-HRMS under full-scan conditions.

The data treatment from suspect screening was done by means of Compound Discoverer 3.3 software where more than 14,234 potential items were reported. The first filtration based on mass accuracy with the unequivocal molecular formula (error in mass within ±2.5 ppm) gave 4372 items (confidence level 4 according to the Schymanski scale [20]). Subsequently, at level-3 confidence, 3620 items were tentatively identified according to MS/MS spectra. After this, the comparison with external and home-made databases reduced the number of tentative identifications at level 2 to 71 for the PLA bag, 52 for the PE bag and 72 for the PLA pellets. Procedural blanks and experimental controls (biotic control without spike) were subtracted from the final peak areas for items identified with a confidence level of 2, resulting in a final list of 23 tentatively identified compounds. Finally, 13 compounds were identified at level 1 and quantified by standard comparison and the external calibration curves. Table 2 lists the additives leached tentatively identified at level-2 confirmation (exact mass with an error within ±2.5 ppm and profile of MS^2^ for the products of molecular ion) from test materials with their temporal evolution throughout the biodegradation process. This evaluation was done by chromatographic peak area obtained at each sampling time compared with the initial peak area. The results shown in Table 2 can be the sum of two processes: the biodegradation of plastic additives and their liberation from the matrix and, second, the generation of degradation products coincident with some plastic additives. In addition, Table 2 compiles the predicted no-effect concentrations (PNECs) of the compounds in freshwater organisms.

To differentiate the main sources of targeted compounds, additional experiments were planned by spiking the mixed liquor with 100 ng/mL of a selected mixture of plastic additives during the same experimental treatment. The main results comparing both types of experiments showed that, for example, the concentration level of azelaic acid increased in both cases, although there was a degree of degradation in the fortified samples during the first week. Therefore, it cannot be stated that the presence of azelaic acid is just because of the leaching from the bioplastic. Similar results were observed for TBEP, Uvinul^®^ 3049, linoleic acid and abietic acid, which is one of the most common plastic additives for bioplastics [22]. This last one was removed after 7 days of biodegradation in spiked experiments, while in the experiments with a commercial PLA bag, it increased during the first seven days, indicating leaching from the biobag. For PLA pellets, the results shown in Table S1 indicate a similar compound profile to that obtained for the PLA bag, with abietic acid, azelaic acid, Uvinul^®^ 3049, and benzoic acid behaving similarly but at higher levels, suggesting leaching as the most feasible mechanism. Finally, in the PE bag experiments, the main compounds released from leaching fossil-based bags were abietic and azelaic acids, as well as TBEP and DEP.

## 3. Discussion

The current study addresses aspects that are largely overlooked in the current literature. Most existing works focus on plastic removal efficiency under different treatments [23,24], the influence of environmental variables such as the number of particles in the removal [25], or the assessment of toxicological effects associated with degradation by-products [26]. In contrast, our study introduces two complementary and innovative components. First, we applied Pyr-GC-HRMS to evaluate the biodegradation of the polymers, providing detailed information about the concentration that is left after the exposure. Second, we simultaneously investigated the leaching of plastic additives during the degradation process through suspect screening analysis by LC-HRMS, enabling an integrated understanding of how polymer breakdown and additive release occur in parallel. This combined analytical approach offers a more comprehensive perspective than previous studies and advances the current knowledge on the environmental behavior of bioplastic materials.

### 3.1. Biodegradation Interpretation

The high degradation percentage of PLA demonstrates the strong capability of CAS microorganisms to degrade this bioplastic. This finding is consistent with the well-documented biodegradability of PLA under specific conditions. Microorganisms present in activated sludge can degrade PLA through the action of both endogenous and exogenous enzymes, including Cytochrome P450. These enzymes facilitate the hydrolysis of PLA into lactic acid monomers, which can subsequently be metabolized by microbial communities [27]. Additionally, the experimental conditions, including a constant pH of 7, controlled temperature, adequate aeration, and the presence of activated sludge, were likely favorable for PLA degradation, as they enhanced microbial activity and promoted efficient enzymatic breakdown. Furthermore, the degree of fragmentation of the PLA bags significantly influenced the degradation rate: smaller fragments provide a higher surface area-to-volume ratio, which facilitates microbial colonization and accelerates the degradation. Finally, the amorphous regions of PLA are more susceptible to hydrolysis than the crystalline regions, which may further contribute to the observed degradation rate [28,29].

When comparing the abiotic degradation of the PLA bag and powder, the degradation of the bags was negligible (0.38%), likely due to the increased resistance imparted by plastic additives commonly used in commercial bags. In contrast, the abiotic control of the PLA powder exhibited an unexpectedly high degradation percentage (56.46%). The main hypothesis is that significant hydrolysis occurred in the water-only controls, as reported in previous studies [29]. Hydrolysis cleaves the ester bonds in PLA, resulting in depolymerization into lactic acid monomers. This abiotic mechanism may therefore account for a substantial portion of the degradation observed in the control group. In addition, PLA pellets typically contain fewer additives than commercial PLA bags, which may have facilitated hydrolytic degradation in the PLA powder. The significant difference between the degradation rates in the experimental and the control groups highlights the crucial role of microbial activity compared with abiotic factors.

Moreover, negligible PE degradation by CAS was obtained, as expected. The high crystallinity and hydrophobic nature of PE confer strong resistance to biodegradation, as its tightly packed crystalline regions limit microbial and enzymatic access [30]. In addition, plastic additives commonly incorporated into commercial PE products further enhance its resistance to degradation [20].

### 3.2. Plastic Additive Leaching

**Figure 1:**
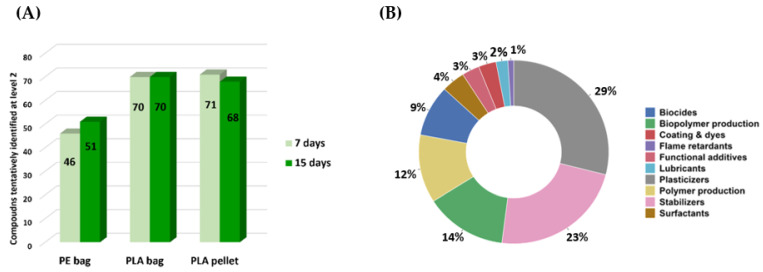
(**A**) Number of plastic additives leached from each material in each experimental time; (**B**) each experimental time and plastic additives family detected in the experiment overall.

Figure 1 shows the number and the type of plastic additives leached from the three materials after 7 and 15 days. Higher amounts of plastic additives were leached from both bioplastics compared to those of fossil-based material (Figure 1A) [31].

First, bioplastics require higher additive levels to emulate the properties of fossil-based materials. Second, the higher biodegradability of bioplastics facilitates the release of additives, since they contain polar functional groups that reduce the interaction strength between the bioplastic matrix and hydrophobic additives (e.g., phthalates), thereby facilitating their migration into the mixed liquor. On the other hand, bioplastics also tend to exhibit lower crystallinity than fossil-based polymers, allowing great mobility of small molecules through the amorphous polymer matrix. Furthermore, their hydrophilic nature promotes water uptake, leading to matrix swelling and enhanced diffusion of additives.

Plasticizers and stabilizers were the most frequently leached families, which is consistent with their widespread use in plastic formulations [32], followed by compounds related to biopolymer synthesis and polymerization precursors.

Despite the advantages of bioplastics in terms of the use of renewable resources and lower carbon footprint, biodegradability, composability of certain bioplastics (e.g., PLA, polyhydroxyalkanoates-PHA), and reduced environmental persistence, the lixiviation of toxic additives should be considered as well. Among the different types of plastics studied in this work, different trends were identified. Compounds that are associated with bioplastic production were only detected in PLA, which had a higher number of polymer-production-related compounds. In finished plastics (both types of bags), flame-retardants such as tris(2-butoxyethyl) phosphate and surfactants used to improve the distribution of other additives, such as pigments, were found, as expected.

On the other hand, plastic additives that were tentatively identified at level 2 included different ultraviolet (UV) absorbers and stabilizers (e.g., benzophenones, plasticizers such as bisphenol A and phthalates) as well as residues of pesticides (e.g., diuron). These tentatively identified compounds are associated with adverse effects on the environment and human health as in, for example, endocrine disruption [28,30,33] or carcinogenicity [34,35].

### 3.3. Environmental Implications

The results from both suspect screening and target analysis (Table 2) were ranked according to their predicted no-effect concentration (PNEC), from lowest to highest and corresponding to increasing potential environmental impact. Grey-shaded cells indicate plastic additives whose concentrations increased over the experimental period in at least one plastic item. Based on PNEC values, the compounds posing the greatest potential environmental risk due to their increasing concentrations and incomplete removal during treatment were the plasticizer trimethylolpropane caprate and the herbicide diuron, both detected at higher levels after treatment in experiments with PLA pellets and PE bags. Other compounds with low PNEC values included nonylbenzene, a plasticizer precursor potentially originating from PLA bag biodegradation, and the antioxidant N-phenyl-1-naphthaleneamine.

Additional compounds of concern for aquatic organisms included pentadecanoic acid and tert-butylperoxy 2-ethylhexyl carbonate, both used in biopolymer production. The plasticizer 2-phenylphenol was detected in PLA pellet experiments after 7 days, while bisphenol A was found across all experiments. Other low-PNEC compounds associated with (bio)polymer synthesis and leached during mixed-liquor treatment included myristoleic acid (pellets); tridecylic acid and 15-hydroxypentadecanoic acid (pellets and PLA bags); 3,9-dodecadiyne, 10-undecenoic acid, and 12-hydroxydodecanoic acid (PLA bags); and 4,4′-dihydroxybiphenyl (all samples).

Several tentatively identified compounds showed increasing concentrations after 7 days, including antioxidants (e.g., 2,6-di-tert-butylphenol, 2,5-di-tert-butylhydroquinone), plasticizers (e.g., diisobutyl phthalate, butylbenzyl phthalate), herbicides and pesticides (e.g., carbetamide, piperonyl butoxide), surfactants and antistatic agents (e.g., lauramide, cetylsulfonic acid), UV stabilizers (e.g., benzophenone, benzotriazole), lubricants (e.g., undecanoic acid), preservatives, softeners, adhesives, corrosion inhibitors, and flame retardants such as tris(2-butoxyethyl) phosphate.

Overall, the experimental results indicate that leachates from biobased plastic materials may pose a higher potential risk to receiving aquatic environments, as reflected by their low PNEC values, consistent with findings reported in previous studies [5,9,10].

## 4. Materials and Methods

### 4.1. Reagents and Standards

The analytical standards of plastic additives triethyl phosphate (TEP), tris(2-butoxyethyl) phosphate (TBEP), tris(2-chloroethyl) phosphate (TCEP), ε-caprolactam, laurolactam, abietic acid, diacetone acrylamide (DAAA), myristic acid, azelaic acid, benzoic acid, and nonanoic acid were purchased from Sigma-Aldrich (Steinheim, Germany). Uvinul^®^ 3049, and Keramide^®^ E Ultra were purchased from AccuStandard (New Haven, CT, USA). Dimethyl phthalate (DMP), dibutyl phthalate (DBP), linoleic acid, oleic acid, palmitic acid and stearic acid were purchased from Fluka (Geneva, Switzerland). Bis-(2-ethylhexyl) phthalate and diethyl phthalate (DEP) were purchased from Riedel-de Haën (Seelze, Germany), and triphenyl phosphate (TPhP) was purchased from Supelco (Bellefonte, PA, USA). Individual stock standard solutions of plastic additives (1000 mg/L) were prepared in methanol.

The analytical standard of polyethylene (PE) (MW~1200 Da) was supplied by Polymer Standard Service GmbH (Mainz, Germany). Polylactic acid (PLA) was purchased from Sigma-Aldrich.

Individual stock standard suspensions of PE and PLA (1000 mg/L) were prepared (10 mg of MPL standard was dispersed in 10 mL toluene), and they were subsequently diluted to obtain the calibration curves for quantification purposes. In all the cases, plastic homogenic suspensions were achieved by using an ultrasonic bath with heating.

All the reagents were of analytical grade. Toluene CHROMASOLV^®^PLUS, which was used as the mobile phase during HPLC analysis, was acquired from Merck (Boston, MA, USA), whereas methanol, acetonitrile, and water were purchased from Fischer Scientific (Loughborough, UK).

GF/F glass microfiber filters with a pore size of 0.70 µm were obtained from Whatman PLC (Maidstone, UK), and nitrogen used as a drying gas with 99.995% purity was supplied by Air Liquide (Barcelona, Spain).

### 4.2. Setup and Sampling

**Figure 2:**
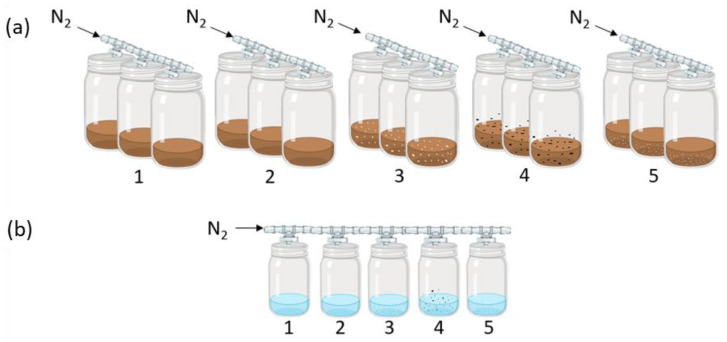
Experiment setup, where in (**a**), 1. sludge control, 2. spiked with plastic additives, 3. PLA bag, 4. PE bag and 5. PLA powder; while in (**b**), 1. water control 2. spiked with plastic additives, 3. PLA bag, 4. PE bag and 5. PLA powder.

Mixed liquor from activated sludge from WWTP was homogenized, and approximately 20 g was weighed and placed in 15 L glass jars for batch experiments. Subsequently, 800 mL of groundwater was added, and the content was vigorously mixed. Parallel control batches containing only groundwater were prepared to serve as abiotic controls and to assess potential adsorption of compounds onto the glass walls. The experimental setup (Figure 2) consisted of jars placed side by side and continuously aerated through the experiment by air bubbling, ensuring aerobic, non-reducing conditions favorable for microbial activity while simultaneously providing mixing.

For each treatment type, three independent biological replicates were prepared, and at each sampling point three analytical replicates were performed for every biological replicate. To evaluate the degradation of plastic additives, three sludge-containing batches and one adsorption control were spiked with the standard mixtures. After 30 min of aeration for homogenization, the first aliquot was collected (time 0). PLA and PE garbage bags were cut into pieces below 5 mm and added separately to the corresponding batch experiments in triplicates of 10 mg/L. PLA pellets were also introduced to separate batch experiments in triplicate at the same concentration. All mixtures were homogenized by air bubbling, and a 100 mL aliquot was collected at time 0.

For batch experiments spiked with plastic additives, 100 mL aliquots were collected at time 0, 7 and 15 days. In contrast, for experiments involving (bio)MPLs, a 100 mL aliquot was taken at time 0 and 500 mL after 15 days (see Table 3).

### 4.3. Sample Pre-Treatment and Extraction

***Plastic additives***. Aliquots of 100 mL were centrifuged (4000 rpm for 5 min at room temperature) and the supernatants were filtered with 0.7 µm glass microfiber filters (Whatman^®^, GF/F grade circles). The extraction and pre-concentration of the samples were done using C18 solid phase extraction (SPE) after comparing different efficiencies of stationary phases for targeted plastic additives. During the comparison, the recovery efficiencies of anionic exchange sorbent (Oasis WAX), cationic exchange sorbent (Oasis WCX) and, finally, hydrophilic-lipophilic balance sorbent (Oasis HLB) were compared. The final procedure was carried out by means of Oasis^®^ WCX 3cc according to the results shown in Table S2 from the Supplementary Materials.

The final protocol included the conditioning of the cartridges with 3 mL of methanol (×2) and 3 mL of HPLC water (×2) under gravity conditions. Then, the samples and the blanks were loaded (100 mL at ca. 1 mL/min using a vacuum pump and further dried for 15 min). Afterwards, the elution of the analytes was made by passing 2.5 mL of methanol (×2) under gravity conditions. Finally, the eluates were evaporated under a nitrogen stream to near dryness and reconstituted with 1 mL of MeOH:H_2_O (1:9). In order to monitor possible cross contamination, a blank sample consisting of 100 mL of HPLC water was extracted using the same procedure in parallel to the rest of the samples.

***(Bio)micronanoplastics.*** The samples taken at time 0 and after 15 days were diluted 1:1 with a saturated solution of NaCl (200 g/L) to facilitate bioMNPL floatability. After NaCl addition, the solution was vigorously homogenized and left undisturbed for 24 h to reach equilibrium. After this time, the samples were centrifuged at 4000 rpm for five minutes at room temperature and the floating MNPLs were filtered through glass microfiber filters with a 0.7 µm pore diameter assisted by a vacuum pump. After filtration, the filters were settled in glass Petri dishes and left overnight to dry at room temperature before extraction. The extraction consisted of solid–liquid extraction using ultrasonic-assisted extraction (USAE) as described elsewhere [36]. The filters were introduced into a glass vial and filled with 10 mL of toluene. Then the extraction was carried out by means of USAE for 10 min and the supernatant was transferred to a glass vial. This procedure was repeated two more times and all of the supernatant (30 mL in total) was evaporated under nitrogen steam to near dryness, transferred to a LC-vial, then further evaporated to a final volume of 1 mL [36].

### 4.4. Instrumental Analysis

The plastic additives were analyzed by liquid chromatography coupled with high-resolution mass spectrometry. This was carried out using an Acquity HPLC (Waters, Milford, MA, USA) system equipped with a reversed-phase column (Purospher^®^ STAR RP-18 endcapped (2 µm) HIBAR^®^ HR 30-2.1 mm). The LC system was coupled with a Q-Exactive^TM^ hybrid quadrupole-Orbitrap^TM^ mass spectrometer (Thermo Fisher Scientific, San Jose, CA, USA) equipped with an electrospray ionization (ESI) source operating in negative and positive modes in two different injections. The mobile phase consisted of water and acetonitrile for the negative acquisition mode, and water with 0.05% formic acid and acetonitrile for the positive acquisition mode (see Table 4). The flow rate was kept at 0.2 mL/min for 15 min, and the sample volume injected was 10 µL. Data acquisition was performed in full-scan mode (100–1500 *m*/*z*) and, in parallel, in data-dependent mode targeting the five most intense ions at a resolution of 70,000 FWHM [3].

The analysis of bioMNPLs was performed using a Trace 1310 series gas chromatograph coupled with a Q-Exactive Orbitrap mass spectrometer (Thermo Scientific, Waltham, MA, USA) equipped with a multi-shot pyrolizer EGA/PY-3030D (Frontier Laboratories, Fukushima, Japan). Pyrolysis was carried out at 600 °C for 0.5 min in single-shot mode. The pyrolizer was connected to a split/spitless injector, operating in split mode. The oven temperature was programmed from 60 °C to 180 °C at 10 °C/min, followed by a ramp at 5 °C/min to 310 °C, which was held for 7 min. The temperature was then decreased to 70 °C at 30 °C/min and maintained for an additional 5 min, resulting in a total run time of 45 min. The injector and pyrolysis interface temperatures were both set to 300 °C. A 30 m × 0.25 mm internal diameter × 0.25 µm film thickness DB-5MS (5% phenyl–95% methyl polysiloxane) fused-silica capillary column (Agilent, Santa Clara, CA, USA) was used to separate the pyro lysates. Ultra-high purity helium (99.9999%) was used as the carrier gas at a constant flow rate of 1 mL/min. The mass spectrometer was equipped with an electron impact (EI) ionization source, working at 70 eV. The acquisition in Orbitrap HRMS was carried out in full-scan mode (70–300 *m*/*z*), working at a resolution of 17,500 FWHM.

### 4.5. Tentative Identification and Quantification of Plastic Additives by LC-HRMS

The data generated from the LC-HRMS was further processed by the specific software Compound Discoverer 3.3, using a slightly modified workflow for environmental analysis data treatment (Environmental Unknown ID using Online and Local Database Searches) with a local database containing more than 500 plastic additives and 45 specifically used for bioplastics. The software was used for data processing and tentative compound identification.

Finally, the compounds tentatively identified at level-2 confidence were quantified when the pure standard was available using external calibration-curve calibration and target analysis by LC-HRMS.

### 4.6. Identification and Quantification of BioMNPLs by Pyr-GC-HRMS

For bioMNPLs, the generated data was processed using Xcalibur 4.1 software (Thermo Fisher Scientific).

**Figure 3:**
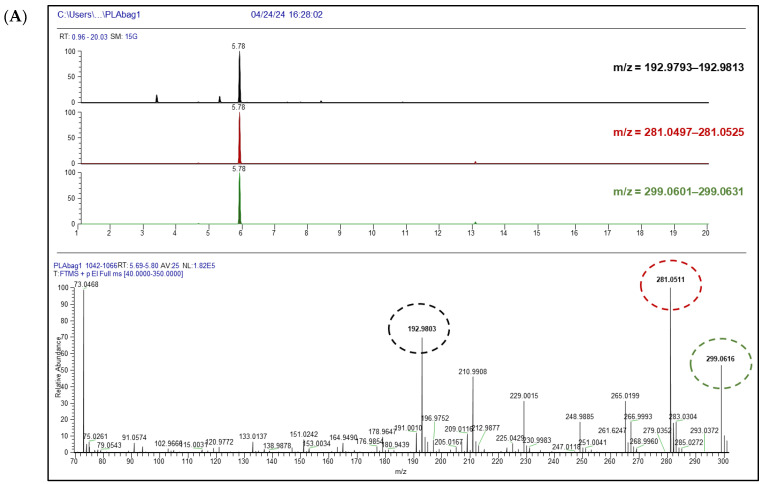

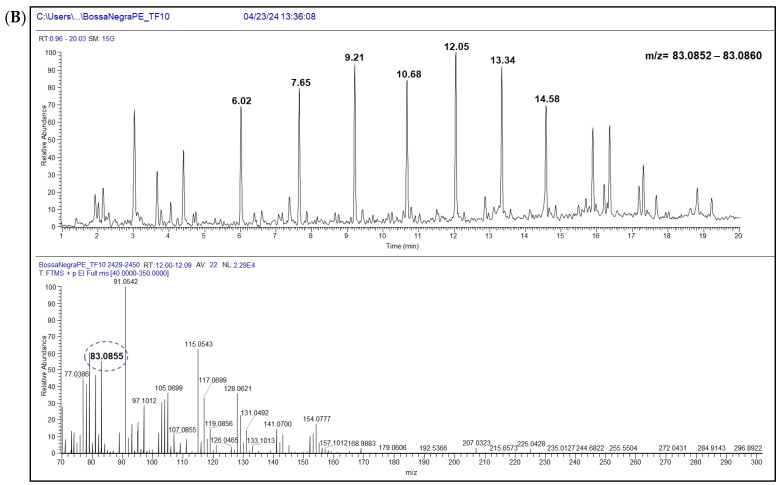
Extracted ion chromatogram (XIC) for (**A**) PLA bag (upper figure) and the corresponding *m*/*z* spectra at a retention time of 5.78 min for the three characteristic *m*/*z* fragments, and (**B**) PE bag (upper figure) with its characteristic *m*/*z*. Results were obtained by Pyr-GC-HRMS analysis.

A homemade library was developed in our research group for the mass spectral loss identification of the most common polymers and biopolymers [4]. The identification during pyrolysis was based on retention time (RT), characteristic *m*/*z* fragment ions and their relative abundance. The identification and quantification of PLA was based on the observation of the most intense ions at an RT of 5.78 min including *m*/*z* 192.9803, 281.0511 and 299.0616, with an error in exact mass within ±2.5 ppm. On the other hand, for the identification of PE, the most intense and repetitive *m*/*z* was 83.0855 at an RT of 6.02, 7.65, 9.21, 10.68, 12.05, 13.34 and 14.58 min. Examples of extracted ion chromatograms (XIC) are shown in Figure 3. The final quantification was done by comparison with external calibration curves, while the percentage of biodegradation was calculated based on the initial experimental concentration and the final experimental concentration after 15 days of treatment.

### 4.7. Quality Assurance and Quality Control

The quality control of the analysis of plastic additives was monitored by analyzing a blank consisting of 100 mL of HPLC water in parallel with each batch of extraction samples. In the instrumental analysis, one blank consisting of the reconstitution mobile phase was injected every three samples. Furthermore, a quality control procedure was used to monitor the traceability of the data generated consisting of a mixed liquor spiked with a mix of plastic additives at 100 ng/mL and analyzed in parallel with the whole batch of samples. The recovery rates were tested in HPLC water fortified with the mixture of compounds from Table S2 using Oasis WCX cartridges in parallel with the rest of the samples to monitor the traceability of the compounds. The results ranged from 49 to 130% with a reproducibility expressed in a relative standard deviation percentage (RSD) below 25%, and with limits of quantification for the target compounds below 1 µg/L.

In the case of bioMNPLs, a blank consisting of 500 mL of HPLC water was filtered. This filter was used as a procedural blank. On the other hand, any possible cross contamination coming from the analysis was monitored by injecting solvent blanks (toluene) every four samples. Also, the traceability of the bioMNPLs was assessed by spiking 500 mL of milliQ water with selected polymers of PE and PLA at 10 mg/L, in triplicate. The recovery rates were between 43 and 50% for PE and between 60 and 71% for PLA, with a reproducibility below 20% (RSD).

To minimize cross-contamination, all laboratory tools and containers used during sample handling were made exclusively of glass. In addition, the personal protective equipment used by laboratory personnel was plastic-free.

## 5. Conclusions

This study provides valuable insights into the factors influencing plastic degradation and underscores the need for sustainable materials and waste management practices. Although the PLA bag showed a high degradation rate under controlled laboratory conditions, these results cannot be directly extrapolated to real WWTP environments, where fluctuating microbial communities, variable physicochemical conditions and operational factors may alter the biodegradation. Furthermore, bioplastics were shown to release a wide range of plastic additives, and conventional treatments can be insufficient for their complete removal; additionally, some additives may pose risks to aquatic organisms. Consequently, further studies are required to assess the proper experimental conditions, as well as to explore the combination of additional treatments such as activated carbon filtration, membrane filtration or advanced oxidation processes to evaluate the elimination of the most persistent additives.

## Figures and Tables

**Table 1:** Biodegradation results of the experiment.

	PE Bag	PLA Bag	PLA Powder
Biodegradation %	18.3 ± 25.8% *	96.8 ± 4.0%	91.3 ± 9.0%

* Variability arises from differences in microbial activity across independent biological replicates.

**Table 2:** The increase or decrease of the detected compounds throughout the experiment was evaluated using the peak areas obtained from the suspect screening analysis. Compounds were tentatively identified at level-2 confidence, together with their corresponding predicted no-effect concentrations (PNECs) for freshwater organisms when available. The temporal trend of each compound is also reported, where ‘-’ denotes absence, ‘↗’ indicates an increase, ‘↗↗’ indicates a further increase relative to the previous time point, ‘=’ denotes no change, and ‘↘’ indicates a decrease. Cells shaded in grey indicate that the presence of the plastic additive increased over the course of the experiment for at least one plastic item.

Compound	Family	PLA Pellet		PLA Bag		PE Bag		PNEC *	
		7d	15d	7d	15d	7d	15d	Conc. (µg/L)	Type
Hexamethoxymethyl melamine	Crosslinking agent	-	-	↗	↗↗	-	-	0.017	Predicted
Allethrin	Insecticide	-	-	↗	-	↗	=	0.024	Experimental
Kadethrin	Insecticide	-	-	↗	-	-	-	0.028	Predicted
TOFA	Biopolymer production	↗	↘	-	-	-	-	0.042	Predicted
Trimethylolpropane caprate	Plasticizer	↗	↗↗	-	-	-	-	0.048	Predicted
Diuron	Herbicide	↗	↗↗	-	-	-	↗	0.049	Experimental
Nonylbenzene	Precursor of plasticizer	-	-	↗	↗↗	-	-	0.049	Predicted
N-Phenyl-1-naphthaleneamine	Antioxidant	↗	=	↗	↗↗	↗	↘	0.060	Predicted
Linoleic acid	Lubricant	-	-	=	↗	-	-	0.077	Predicted
Dodecyl isocyanate	Polymer production	-	-	↗	↘	-	-	0.10	Predicted
Ethyl myristate	Bio-based plasticizer	↗	-	↗	-	-	-	0.14	Predicted
Pentadecanoic acid	Biopolymer production	↗	↗↗	-	-	-	-	0.16	Predicted
2-Phenylphenol	Fungicide	↗	↗↗	-	-	-	-	0.18	Experimental
Ricinoleic acid	Biopolymer production	-	-	↗	-	-	-	0.18	Predicted
Mono(2-ethylhexyl) phthalate	Plasticizer	↗	↘	↗	=	↗	=	0.19	Predicted
Tert-Butylperoxy 2-ethylhexyl carbonate	Polymerization initiator	↗	↘	↗	↗↗	↗	-	0.19	Predicted
Lauryl acrylate	Coatings	↗	↘	↗	↘	↗	↘	0.20	Predicted
Bisphenol A	Plasticizer/Epoxy resins	↗	↗↗	↗	↗↗	↗	↗↗	0.24	Experimental
16-Hydroxyhexadecanoic acid	Biopolymer production	↗	=	↗	-	-	-	0.29	Predicted
Myristoleic acid	Biopolymer production	↗	↗↗	-	-	-	-	0.29	Predicted
Sethoxydim	Herbicide	↗	↘	-	-	↗	-	0.32	Experimental
2,6-di-tert-Butylphenol	Antioxidant	↗	↗↗	↗	↘	↗	-	0.36	Predicted
Dodecenoic acid	Biopolymer production	↗	↘	-	-	-	-	0.36	Predicted
Carbetamide	Herbicide	-	-	-	↗			0.37	Experimental
2,6-di-tert-Butyl-4-methoxyphenol	Antioxidant	↗	↗↗	-	-	-	-	0.40	Predicted
3,9-Dodecadiyne	Polymer production	-	-	↗	↗↗	-	-	0.41	Predicted
Lauramide	Surfactant	-	-	-	-	-	↗	0.44	Predicted
Cetylsulfonic acid	Antistatic agent	-	-	-	-	-	↗	0.46	Predicted
Tridecylic acid	Biopolymer production	↗	↗↗	↗	=	-	-	0.54	Predicted
15-Hydroxypentadecanoic acid	Biopolymer production	↗	↗↗	-	↗	-	-	0.58	Predicted
Bis(2-ethylhexyl) adipate	Plasticizer	-	-	↗	=	↗	-	0.70	Experimental
Uvinul 3049	UV stabilizer	-	↗	↗	↗↗	-	-	0.73	Predicted
2,5-di-tert-Butylhydroquinone	Antioxidant	↗	↗↗	↗	↗↗	↗	↗↗	0.89	Predicted
2-Hydroxymyristic acid	Biopolymer production	↗	=	-	-	-	-	1.11	Predicted
Diisobutylphthalate	Plasticizer	-	-	↗	↗↗	↗	↘	1.11	Predicted
3,5-di-tert-Butyl-4-hydroxybenzoic acid	Antioxidant	-	↗	↗	=	↗	↗↗	1.13	Predicted
Piperonyl-butoxide	Pesticide synergist	↗	=	↗	↗↗	↗	=	1.20	Experimental
Bis(2-ethylhexyl) phthalate	Plasticizer	=	=	=	=	=	=	1.30	Experimental
Laurylbetaine	Surfactant	-	-	-	↗	-	-	1.57	Predicted
Lauric acid	Surfactant	↗	=	↗	=	↗	↗↗	1.58	Experimental
Undecanoic acid	Lubricant	↗	=	-	-	↗	↗↗	1.74	Predicted
Valerophenone	Photoinitiator UV	-	-	-	↗	-	-	2.09	Predicted
Fenobucarb	Insecticide	↗	=	↗	=	↗	↘	2.13	Predicted
Benzanilide	UV stabilizer	↗	↗↗	↗	↘	↗	=	2.14	Predicted
Abietic acid	Tackifier	↗	↘	↗	↘	↗	↘	2.20	Predicted
10-Undecenoic acid	Biopolymer production	↗	=	↗	↗↗	-	-	2.26	Predicted
Monobutyl phthalate	Plasticizer	↗	=	-	-	↗	=	2.31	Predicted
4-Methylbenzophenone	UV stabilizer	↗	=	↗	↗↗	↗	↘	2.34	Predicted
Diethyl sebacate	Plasticizer	↗	↗↗	↗	↗↗	↗	↗↗	2.57	Predicted
4-Hydroxybenzophenone	UV stabilizer	↗	↗↗	↗	↘	↗	↗↗	2.77	Predicted
Ametoctradin	Fungicide	↗	↘	↗	-	↗	↘	4.40	Experimental
Tetradecanedioic acid	Biopolymer production	↗	↘	↗	=	-	-	4.66	Predicted
4-Nitrophenol	Polymer production	-	-	-	↗	-	-	5.00	Experimental
Butylbenzylphthalate	Plasticizer	↗	↗↗	-	↗	-	↗	5.20	Experimental
4,4′-Dihydroxybiphenyl	Polymer production	-	-	↗	↗↗	-	↗	5.23	Predicted
Benzophenone	UV stabilizer	↗	↗↗	-	↗	-	↗	5.40	Experimental
4-tert-butylphenol	Adhesive	↗	↗↗	-	-	-	↗	5.41	Predicted
Bisphenol F	Plasticizer/Epoxy resins	↗	↘	↗	-	↗	↘	5.44	Predicted
Decanoic acid	Lubricant	↗	↘	↗	-	↗	=	5.70	Experimental
12-Hydroxydodecanoic acid	Biopolymer production	↗	↘	↗	↗↗	-	-	6.86	Predicted
Diethyl azelate	Plasticizer	↗	-	↗	-	↗	=	7.47	Predicted
4-Methylbenzotriazole	Corrosion inhibitor	↗	↗↗	↗	↗↗	↗	↗↗	8.00	Experimental
2-Methyl-1,2-benzothiazol-3(2H)-one	Preservative	↗	↗↗	↗	↗↗	↗	↗↗	8.14	Predicted
n-Butyl methacrylate	Coatings	-	-	↗	-	↗	-	9.33	Predicted
27-Methyl-2,5,8,11,14,17,20,23,26-nonaoxaoctacosane	Softener	↗	↗↗	↗	↗↗	-	-	10.8	Predicted
2,4,6-Trimethylphenol	Antioxidant	↗	↗↗	↗	-	-	↗	12.3	Predicted
Dodecanedioic acid	Corrosion inhibitor	-	-	-	↗	-	-	12.4	Predicted
2-Hydroxybenzothiazole	Preservative	-	-	↗	↗↗	↗	↗↗	14.0	Experimental
Furmecyclox	Fungicide	-	-	-	↗	-	-	15.1	Predicted
11-Hydroxyundecanoic acid	Biopolymer production	↗	↘	-	-	-	-	17.4	Predicted
Benzotriazole	UV stabilizer	↗	↘	↗	=	↗	↘	19.0	Experimental
N-butylbenzenesulfonamide	Plasticizer	-	-	↗	=	-	-	21.1	Predicted
Tris(2-butoxyethyl) phosphate	Flame retardant	-	-	↗	↗↗	↗	↘	24.0	Experimental
21-Methyl-2,5,8,11,14,17,20-heptaoxadocosane	Surfactant	-	↗	-	-	-	↗	32.4	Predicted
3-Hydroxydecanoic acid	Biopolymer production	-	-	↗	↗↗	-	-	33.6	Predicted
1,3-Divinyl-2-imidazolidinone	Crosslinking agent	-	↗	-	↗	-	-	34.2	Predicted
Phthalic anhydride	Monomer for plasticizers	-	-	-	-	↗	-	36.7	Predicted
Benzoic acid	Preservative	-	↗	-	-	-	-	44.6	Experimental
Heptaethylene glycol	Plasticizer	-	-	-	↗	-	-	47.4	Predicted
PPG n4	Plasticizer	↗	↘	↗	-	↗	↗↗	69.4	Predicted
Diethylphthalate	Plasticizer	-	-	-	-	↗	↘	73.0	Experimental
14-hydroxy-3,6,9,12-tetraoxatetradecyl acrylate	Monomer for UV-curable plastics	-	-	↗	↗↗	-	-	81.1	Predicted
Benzenesulfonamide	Plasticizer	-	-	↗	=	-	-	81.9	Predicted
DEET	Insecticide	-	↗	-	-	↗	↗↗	88.0	Experimental
4-Methylphenol	Antioxidant precursor	-	-	↗	↗↗	-	↗	100	Experimental
4-Toluenesulfonamide	Plasticizer	↗	-	-	-	-	-	150	Experimental
1,1′-(1,12-Dodecanediyl) di (2,4, -imidazolidinedione)	Curing agent	↗	↗↗	-	↗	-	-	n.a.	
1,4-Diamino-2,3-bis(4-nonylphenoxy)-9,10-anthraquinone	Dyes and pigments in plastics	-	-	-	-	↗	↗↗	n.a.	
2,5,8,11,14-Pentaoxahexadecane	Dispersing agent	↗	↗↗	↗	↗↗	-	-	n.a.	
2-[(1E)-1-Nonen-1-yl]succinic acid	Biopolymer production	-	-	↗	↗↗	-	-	n.a.	
23-Hydroxy-3,6,9,12,15,18,21-heptaoxatricos-1-yl nonanoate	Surfactant	↗	↗↗	↗	↗	-	↗	n.a.	
23-Hydroxy-3,6,9,12,15,18,21-heptaoxatricos-1-yl octanoate	Surfactant	↗	=	↗	↘	-	-	n.a.	
29-Hydroxy-3,6,9,12,15,18,21,24,27-nonaoxanonacos-1-yl decanoate	Surfactant	↗	↗↗	-	-	-	-	n.a.	
2-Dodecanoyl-1,2-benzathiazol-3(2H)-one 1,1-dioxide	Preservative	↗	↘	-	-	-	-	n.a.	
2-Hydroxy-2-(2-isopropoxy-2-oxoethyl)succinate	Biopolymer production	↗	-	↗	-	-	-	n.a.	
4-Acetamido-Tempo	Polymer production	↗	↘	-	-	-	-	n.a.	
4-Biphenylyl isocyanate	Curing Agent	-	-	-	-	↗	↘	n.a.	
4-Hydroxyphenyl 4-hydroxybenzoate	Plasticizer	↗	↗↗	↗	↗↗	↗	↗↗	n.a.	
6-(Nonanoylamino)hexanoic acid	Biopolymer production	-	-	↗	=	-	-	n.a.	
Acetamiprid-metabolite-IM-2-1	Pesticide metabolite	↗	↘	-	-	-	-	n.a.	
Azelaic acid	Plasticizer	↗	↗↗	↗	↗↗	↗	↗↗	n.a.	
Bis(2-ethylhexyl) carbonoperoxoate	Polymerization initiator	↗	↘	↗	↘	↗	↘	n.a.	
Diethanolamine laurate	Surfactant	-	-	↗	-	↗	↗↗	n.a.	
Heptadecanedioic acid	Biopolymer production	↗	-	↗	↘	-	-	n.a.	
N-Methylhexadecanamide	Slip Agent	-	-	↗	↗↗	↗	↘	n.a.	
Octyl 4-methylbenzenesulfonate	Surfactant	-	-	↗	↘	-	-	n.a.	
Pentadecanedioic acid	Biopolymer production	↗	↘	↗	=	-	-	n.a.	
PPG n6	Plasticizer	-	-	-	↗	↗	-	n.a.	
PPG n7	Plasticizer	-	-	-	-	↗	-	n.a.	
PPG n8	Plasticizer	-	-	-	-	↗	-	n.a.	
PPG n9	Plasticizer	-	-	-	-	↗	↘	n.a.	
PEG n11	Plasticizer	↗	↘	-	-	-	-	n.a.	
PEG n12	Plasticizer	↗	↗	↗	↗↗			n.a.	
PEG n13	Plasticizer	↗	↗	↗	↗↗	-	↗	n.a.	
PEG n14	Plasticizer	↗	=	↗	↘	-	↗	n.a.	
PEG n15	Plasticizer	↗	↗	↗	=	-	↗	n.a.	
PEG n16	Plasticizer	↗	↘	↗	↘	-	-	n.a.	
β-methylstyrene	Polymer production	-	-	↗	↘	-	-	n.a.	

* **PNEC:** Predicted no-effect concentration—values extracted from NORMAN database [21]; **n.a.:** not applicable; no values reported in the literature.

**Table 3:** Summary of the experimental setup and sampling days.

	Spiking Level	Analysis Type		
		t = 0	t = 7 Days	t = 15 Days
Control	-	Plastic additives + MNPLs	Plastic additives	Plastic additives + MNPLs
Plastic additives	10 mg/L	-	Plastic additives	Plastic additives
PLA bag	10 mg/L	-	Plastic additives	Plastic additives + MNPLs
PE bag	10 mg/L	-	Plastic additives	Plastic additives + MNPLs
PLA powder	10 µg/L	-	Plastic additives	Plastic additives + MNPLs

**Table 4:** Liquid chromatography gradient for the separation of plastic additives.

Time	%A (Aqueous Phase)	%B (Organic Phase)
**0**	90	10
**5**	90	10
**10**	50	50
**12**	10	90
**14**	90	10
**15**	90	10

## Data Availability

The original contributions presented in this study are included in the article/Supplementary Materials. Further inquiries can be directed to the corresponding authors.

## Supplementary Materials

The following supporting information can be downloaded at: https://www.mdpi.com/article/10.3390/molecules31111878/s1, Table S1: Concentration (µg/L) of plastic additives leached from the different plastic items over experimental time; Table S2: Relative abundances (%) of each compound in different SPE cartridges; Table S3: Supporting information (excel format): suspect screening results.

## References

[1] Plastics Europe Plastics Europe Launches Plastics—The Fast Facts. https://plasticseurope.org/media/plastics-europe-launches-the-plastics-the-fast-facts-2023/.

[2] North E.J., Halden R.U. (2013). Plastics and environmental health: The road ahead. Rev. Environ. Health.

[3] Ali S.S., Abdelkarim E.A., Elsamahy T., Al-Tohamy R., Li F., Kornaros M., Zuorro A., Zhu D., Sun J. (2023). Bioplastic production in terms of life cycle assessment: A state-of-the-art review. Environ. Sci. Ecotechnol..

[4] Llorca M., Álvarez-Muñoz D., Ábalos M., Rodríguez-Mozaz S., Santos L.H.M.L.M., León V.M., Campillo J.A., Martínez-Gómez C., Abad E., Farré M. (2020). Microplastics in Mediterranean coastal area: Toxicity and impact for the environment and human health. Trends Environ. Anal. Chem..

[5] Vega-Herrera A., Llorca M., Savva K., León V., Abad E., Farré M. (2021). Screening and Quantification of Micro(Nano)Plastics and Plastic Additives in the Seawater of Mar Menor Lagoon. Front. Mar. Sci..

[6] Bahl S., Dolma J., Jyot Singh J., Sehgal S. (2021). Biodegradation of plastics: A state of the art review. Mater. Today Proc..

[7] Roy Chong J.W., Tan X., Khoo K.S., Ng H.S., Jonglertjunya W., Yew G.Y., Show P.L. (2022). Microalgae-based bioplastics: Future solution towards mitigation of plastic wastes. Environ. Res..

[8] European Bioplastics e.V. Bioplastics Market Development Update. https://www.european-bioplastics.org/bioplastics-market-development-update-2023-2/.

[9] Savva K., Borrell X., Moreno T., Pérez-Pomeda I., Barata C., Llorca M., Farré M. (2023). Cytotoxicity assessment and suspected screening of PLASTIC ADDITIVES in bioplastics of single-use household items. Chemosphere.

[10] Savva K., Farré M., Barata C. (2023). Sublethal effects of bio-plastic microparticles and their components on the behaviour of Daphnia magna. Environ. Res..

[11] Piyathilake U., Lin C., Bolan N., Bundschuh J., Rinklebe J., Herath I. (2024). Exploring the hidden environmental pollution of microplastics derived from bioplastics: A review. Chemosphere.

[12] Shamsuddin I.M., Jafar J., Shawai A.S.A., Yusuf S., Lateefah M., Aminu I.M. (2017). Bioplastics as Better Alternative to Petroplastics and Their Role in National Sustainability: A Review. Arch. Biochem. Biophys..

[13] Knowledge 4 Policy—European Comission BKfp. https://knowledge4policy.ec.europa.eu/glossary-item/biodegradability_en.

[14] Tabone M.D., Cregg J.J., Beckman E.J., Landis A.E. (2010). Sustainability Metrics: Life Cycle Assessment and Green Design in Polymers. Environ. Sci. Technol..

[15] Pinto J., Dias M., Amaral J., Ivanov M., Paixão J.A., Coimbra M.A., Ferreira P., Pereira E., Gonçalves I. (2022). Influence of UV degradation of bioplastics on the amplification of mercury bioavailability in aquatic environments. Mar. Pollut. Bull..

[16] Panepinto D., Fiore S., Zappone M., Genon G., Meucci L. (2016). Evaluation of the energy efficiency of a large wastewater treatment plant in Italy. Appl. Energy.

[17] Schirinzi G.F., Llorca M., Seró R., Moyano E., Barceló D., Abad E., Farré M. (2019). Trace analysis of polystyrene microplastics in natural waters. Chemosphere.

[18] Llorca M., Vega-Herrera A., Schirinzi G., Savva K., Abad E., Farré M. (2021). Screening of suspected micro(nano)plastics in the Ebro Delta (Mediterranean Sea). J. Hazard. Mater..

[19] Mistry A.N., Kachenchart B., Wongthanaroj A., Somwangthanaroj A., Luepromchai E. (2022). Rapid biodegradation of high molecular weight semi-crystalline polylactic acid at ambient temperature via enzymatic and alkaline hydrolysis by a defined bacterial consortium. Polym. Degrad. Stab..

[20] Schymanski E.L., Jeon J., Gulde R., Fenner K., Ruff M., Singer H.P., Hollender J. (2014). Identifying Small Molecules via High Resolution Mass Spectrometry: Communicating Confidence. Environ. Sci. Technol..

[21] NORMAN Database System. https://www.norman-network.com/nds/.

[22] Pérez-Albaladejo E., Solé M., Porte C. (2020). Plastics and plastic additives as inducers of oxidative stress. Curr. Opin. Toxicol..

[23] García-Depraect O., Lebrero R., Rodriguez-Vega S., Bordel S., Santos-Beneit F., Martínez-Mendoza L.J., Börner R.A., Börner T., Munoz R. (2022). Biodegradation of bioplastics under aerobic and anaerobic aqueous conditions: Kinetics, carbon fate and particle size effect. Bioresour. Technol..

[24] Cucina M., Carlet L., De Nisi P., Somensi C.A., Giordano A., Adani F. (2022). Degradation of biodegradable bioplastics under thermophilic anaerobic digestion: A full-scale approach. J. Clean. Prod..

[25] Wei W., Huang Q.-S., Sun J., Dai X., Ni B.-J. (2019). Revealing the mechanisms of polyethylene microplastics affecting anaerobic digestion of waste activated sludge. Environ. Sci. Technol..

[26] Mut N.N.N., Na J., Nam G., Jung J. (2024). The biodegradation of polylactic acid microplastic and their toxic effect after biofouling in activate sludge. Environ. Pollut..

[27] Garlotta D. (2001). A Literature Review of Poly(Lactic Acid). J. Polym. Environ..

[28] Tokiwa Y., Calabia B.P. (2006). Biodegradability and biodegradation of poly(lactide). Appl. Microbiol. Biotechnol..

[29] Zhang Y., Pedersen J.N., Eser B.E., Guo Z. (2022). Biodegradation of polyethylene and polystyrene: From microbial deterioration to enzyme discovery. Biotechnol. Adv..

[30] Ojeda T.F.M., Dalmolin E., Forte M.M.C., Jacques R.J.S., Bento F.M., Camargo F.A.O. (2009). Abiotic and biotic degradation of oxo-biodegradable polyethylenes. Polym. Degrad. Stab..

[31] Boldrini A., Gaggelli N., Falcai F., Polvani A., Talarico L., Galgani L., Cirrone R., Liu X., Loiselle S. (2024). Emerging Contaminants from Bioplastic Pollution in Marine Waters. Water.

[32] Kim S., Choi K. (2014). Occurrences, toxicities, and ecological risks of benzophenone-3, a common component of organic sunscreen products: A mini-review. Environ. Int..

[33] Mao J., Jain A., Denslow N.D., Nouri M.-Z., Chen S., Wang T., Zhu N., Koh J., Sarma S.J., Sumner B. (2020). Bisphenol A and bisphenol S disruptions of the mouse placenta and potential effects on the placenta–brain axis. Proc. Natl. Acad. Sci. USA.

[34] Huovinen M., Loikkanen J., Naarala J., Vähäkangas K. (2015). Toxicity of diuron in human cancer cells. Toxicol. Vitr..

[35] Benson R. (2009). Hazard to the developing male reproductive system from cumulative exposure to phthalate esters—Dibutyl phthalate, diisobutyl phthalate, butylbenzyl phthalate, diethylhexyl phthalate, dipentyl phthalate, and diisononyl phthalate. Regul. Toxicol. Pharmacol..

[36] Garcia-Torné M., Abad E., Almeida D., Llorca M., Farré M. (2023). Assessment of Micro- and Nanoplastic Composition (Polymers and Additives) in the Gastrointestinal Tracts of Ebro River Fishes. Molecules.

