# Supplementary material for: Evaluation of the Efficiency of Biological Treatment in Activated Sludge from a WWTP at Laboratory Scale for the Elimination of Biomicroplastics and Related Products
Source: Molecules. 2026 May 29;31(11):1878. doi: 10.3390/molecules31111878 (PMC13258774; doi:10.3390/molecules31111878)
Supplement: Supplementary file 1 [file molecules-31-01878-s001.zip › Tables S1 and S2.pdf]

Supplementary Material

Evaluation of the Efficiency of Biological Treatment in Activated Sludge from a WWTP at Laboratory Scale for the Elimination of Biomicroplastics and Related Products

David Alcaide-Benavides [1,2], Eloy Torres-Arévalo [1,2], Marinella Farré [1,*] and Marta Llorca [1,*]

[1] Institute of Environmental Assessment and Water Research, C/Jordi Girona, 18-26, 08034 Barcelona, Spain; dabqsh@cid.csic.es (D.A.-B.)
[2] Doctoral Program in Analytical Chemistry and Environmental Science, Department of Chemical Engineering and Analytical Chemistry, University of Barcelona, 08028 Barcelona, Spain
* Correspondence: mfuqam@cid.csic.es (M.F.); mlcqam@cid.csic.es (M.L.)

Table S1. Concentration (µg/L) of plastic additives leached from the different plastic items along experimental time.

	T0	PLA bag		PLA pellet		PE bag	
		**T7**	**T15**	**T7**	**T15**	**T7**	**T15**
**4-Methyl-1H-Benzotriazole**	n.d	<LOQ	0.013	0.003	0.005	<LOQ	0.005
**Abietic acid**	0.094	0.107	0.097	0.105	0.096	0.107	0.096
**Azelaic acid**	<LOQ	0.010	0.268	0.034	0.086	<LOQ	0.062
**Benzoic acid**	2.43	1.31	1.53	1.47	2.91	1.36	1.70
**Benzophenone**	n.d	n.d	1.10	0.949	1.05	n.d	0.781
**Benzotriazole**	n.d	<LOQ	<LOQ	0.008	0.002	0.003	0.002
**Bisphenol A**	<LOQ	0.054	4.07	2.87	4.05	1.72	4.79
**Bis(2-ethylhexyl) phthalate**	<LOQ	<LOQ	<LOQ	<LOQ	<LOQ	<LOQ	<LOQ
**Diethyl phthalate**	2.730	<LOQ	<LOQ	<LOQ	<LOQ	3.95	1.22
**Linoleic acid**	<LOQ	<LOQ	0.014	<LOQ	<LOQ	<LOQ	<LOQ
**p-toluenesulfonamide**	n.d	n.d	n.d	0.048	n.d	n.d	n.d
**Tris(2-butoxyethyl) phosphate**	0.0018	0.018	0.0207	0.0019	0.0019	0.023	0.020
**Uvinul 30/49**	<LOQ	<LOQ	<LOQ	n.d	<LOQ	n.d	n.d

n.d.: not detected

<LOQ: below limit of quantification 1 µg/L

Table S2. Relative abundances (%) of each compound in different SPE cartridges.

Compound	HLB	WAX	WCX
**2,6-di-tert-butylphenol**	1	1	103
**4-Methyl-1H-Benzotriazole**	100	85	95
**Abietic acid**	96	6	97
**Accelerator BBTS**	0	100	0
**Azelaic acid**	100	1	98
**Benzoic acid**	61	0	105
**Benzophenone**	100	8	83
**Benzotriazole**	97	80	100
**Bis(ethylhexyl)phthalate**	53	49	101
**Bisphenol A**	40	89	97
**Bisphenol S**	80	100	99
**Caprolactam**	100	85	95
**Citroflex 4**	6	0	110
**Diacetone acrylamide**	100	41	90
**Diethyl phthalate**	52	0	99
**Dimethyl adipate**	100	70	78
**Dimethyl phthalate**	100	32	59
**Dipropylene glycol dimethyl ether**	0	0	101
**Kemamide E Ultra**	41	0	96
**Laurolactam**	93	84	97
**Linoleic acid**	33	14	100
**Myristic acid**	22	8	104
**Nonanoic acid**	13	15	103
**p-toluenesulfonamide**	94	94	94
**Santoflex**	44	100	0
**Stearic acid**	10	9	100
**Triethyl phosphate**	91	26	91
**Triphenyl phosphate**	27	100	73
**Tris(2-butoxyethyl) phosphate**	99	92	105
**Tris(2-chloroethyl) phosphate**	99	92	96
**Uvinul 30/49**	7	92	95
